# Low cervical vertebral CT value increased early subsidence of titanium mesh cage after anterior cervical corpectomy and fusion

**DOI:** 10.1186/s13018-022-03239-6

**Published:** 2022-07-16

**Authors:** Zhiqiang Wang, Jun Mei, Xiaoning Feng, Chen Deng, Xuefeng Tian, Junqiao Lv, Lin Sun

**Affiliations:** 1 Third Hospital of Shanxi Medical University, Shanxi Bethune Hospital, Shanxi Academy of Medical Sciences, Tongji Shanxi Hospital, Taiyuan, 030032 China; 2 Department of orthopedics, Shanxi Bethune Hospital, Shanxi Academy of Medical Sciences, Tongji Shanxi Hospital, Third Hospital of Shanxi Medical University, Taiyuan, 030032 China; 3 Tongji Hospital, Tongji Medical College, Huazhong University of Science and Technology, Wuhan, 430030 China

**Keywords:** Computed tomography, Hounsfield units, Anterior cervical corpectomy and fusion, Titanium mesh cage, Subsidence

## Abstract

**Study design:**

This study was a retrospective review.

**Objective:**

To study the predictive effect of Hounsfield units (HU) value in the cervical vertebral body derived from computed tomography (CT) on the early titanium mesh cage (TMC) subsidence after anterior cervical corpectomy and fusion (ACCF).

**Methods:**

This retrospective study was conducted on patients who underwent ACCF at one institution between January 2014 and December 2018. We collected date included age, gender, body mass index (BMI), disease type, surgical segment, whether merge ACDF, HU value of the vertebral body and endplate, vertebral body height loss, cervical lordosis angle, and cervical sagittal alignment. VAS, JOA, and NDI were used to assess clinical efficacy. Univariate analysis was performed to screen the influencing factors of TMC subsidence, and then logistic regression was used to find out the independent risk factors. The ROC curve and area under curve (AUC) were used to analyze the HU value to predict the TMC subsidence.

**Results:**

A total of 85 patients who accepted ACCF were included in this study, and early titanium mesh cage subsidence was demonstrated in 29 patients. The subsidence rate was 34.1%. The JOA, VAS, and NDI scores significantly improved in both groups after the operation. Between the subsidence and non-subsidence groups, there were significant differences in age, intervertebral distraction height, and HU value in both upper and lower vertebral body and endplate. The logistic regression analysis proved that the HU value of the lower vertebral body was an independent risk of TMC subsidence, the AUC was 0.866, and the most appropriate threshold of the HU value was 275 (sensitivity: 87.5%, specificity: 79.3%).

**Conclusion:**

Preoperative cervical CT value is an independent correlative factor for early TMC subsidence after ACCF, and patients with a low CT value of the inferior vertebral body of the operative segment have a higher risk of TMC subsidence in the early postoperative period.

*Trial registration*: This study is undergoing retrospective registration.

## Introduction

Anterior cervical corpectomy and fusion (ACCF) is a common surgical method for patients with degenerative cervical spinal diseases [1]. At present, a titanium mesh cage (TMC) with autogenous bone is the main choice of an interbody fusion device in ACCF [2, 3]. It has the advantages of sufficient decompression, good biocompatibility, high bone graft fusion rate, and immediate stability after surgery, and has good therapeutic effects [4–6]. However, there have been more and more reports on its complications, especially postoperative TMC subsidence, which results in changes in cervical curvature and vertebral height. In severe cases, it could cause neck and shoulder pain and aggravate the symptoms of spinal cord compression [7, 8]. Therefore, effective interventions to prevent TMC subsidence should be developed. The stability of TMC is related to the bone mineral density (BMD) of the cervical vertebral body, and the reduction of bone density will lead to a decrease in the bearing capacity of the cervical vertebral body, so patients with severe osteoporosis are prone to TMC subsidence [9].

Dual-energy X-ray absorptiometry (DXA) is currently the gold standard for measuring BMD and diagnosing osteoporosis [10]. However, preoperative DXA scans are not routinely obtained for patients with cervical spine degenerative diseases, and the BMD is measured in the lumbar spine, which cannot directly reflect the bone condition of the cervical spine [11–13]. As a bone quality assessment, the measurement of Hounsfield units (HU) value can be completed by computerized tomography (CT) examination, without additional imaging examination, and it is easy to obtain [14]. Several reports have shown that the HU value of the vertebral was closely related to BMD and could evaluate the risk of pedicle screw loosening and cage subsidence in lumbar spine surgery [15–18]. A few studies have evaluated the association between vertebral body HU value and cage subsidence after anterior cervical discectomy and fusion (ACDF) [19, 20]. However, the relationship between the HU value of the vertebral body and TMC subsidence in ACCF remains unclear.

In this study, we measured the HU value based on preoperative cervical CT in patients, analyzed the clinical and radiologic evaluation of TMC subsidence, and evaluated the relationship between cervical vertebral body HU value and TMC subsidence in the early stage after ACCF.

## Methods

### Patient population

This study was approved by the Ethical Committee of our hospital and each patient signed the informed consent. We reviewed patients undergoing ACCF by a single surgical team between January 2014 and December 2018 at our orthopedic department. The inclusion criteria were as follows: (1) a definite diagnosis of cervical spondylosis; (2) follow-up data for at least 12 months with radiographs; (3) accept ACCF surgery by the same team of spine surgeons; (4) patients with preoperative spine CT and X-ray within 1 week before surgery. The exclusion criteria were as follows: (1) patients with spine infection, spine tumor, spine trauma, and metabolic bone disease; (2) patients with endplate damage or incompleteness; (3) long-term use of hormones or combined with immune diseases; and (4) incomplete radiologic data or functional score data.

### Patient and surgical factors

As potential contributing factors for TMC subsidence, we collected sex, age, BMI, diabetes, hypertension, coronary heart disease, and disease type. In addition to patient information, we collected data on the operation, such as surgical segment, merge ACDF, hospital stay, surgery time, and blood loss.

### Surgical procedure

All operations were performed by the same surgical team. After anesthesia, the patient was placed in a supine position with mild hyperextension of the neck. A transverse incision was made anterior to the right neck, and the approach was made between the carotid sheath and the visceral sheath to expose the cervical spine. Determine the surgical segment by G-arm fluoroscopy, install a spacer, remove the nucleus pulposus and annulus fibrosus with a spatula, and remove the cartilage endplate to the point of bleeding. Part of the vertebral body of the responsible segment was removed to reach the dura mater, and the compression of the dura mater and the nerve root was completely relieved. After the distractor was released, the appropriate TMC was selected according to the scope of resection, filled with autologous cancellous bone fragments, and then implanted into the bone groove. The location of the cage was confirmed using G-arm fluoroscopy. Then, install a suitable length of a titanium plate in front of the cervical vertebral. The incision was rinsed, negative pressure drainage was placed, the incision was sutured layer by layer, and the operation was completed.

### HU measurements

**Fig. 1:**
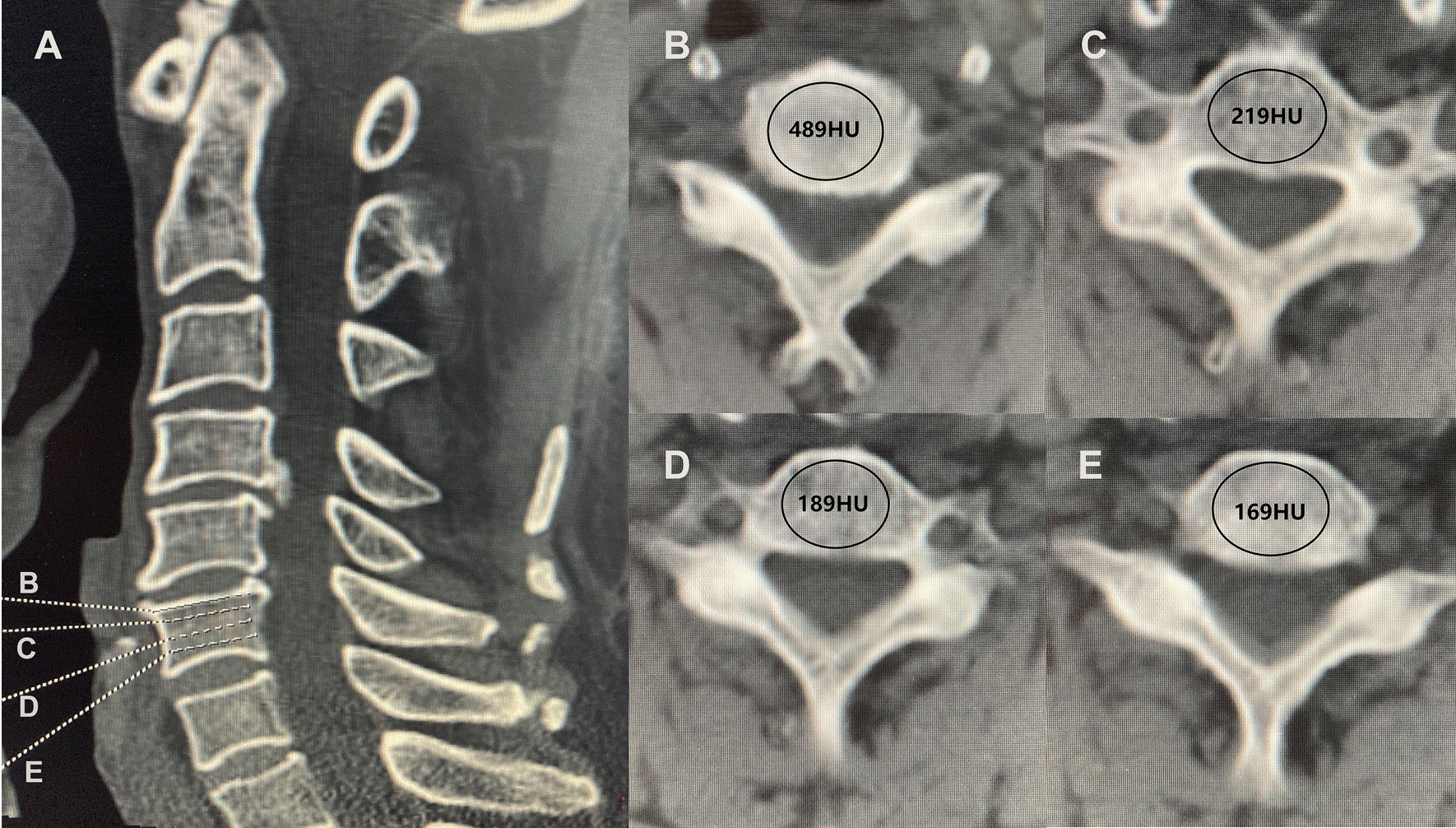
Mid-sagittal (**A**) and axial CT images demonstrating the measurement of endplate (**B**) and vertebral body (**C**–**E**) HU value

Before surgery, all the patients underwent three-dimensional reconstructive cervical CT (PHILIPS, Brilliance, tube voltage 120 kV). Then, the picture archiving and communication system (PACS) was used to calculate the HU value automatically. HU values were measured using CT scans according to a previously described method [14]. The endplate HU value was calculated as the region of interest (ROI) of the upper and lower endplate. The average HU values of each vertebral body based on the axial plane inferior only to the superior endplate, middle of the vertebral body, and axial plane superior only to the inferior endplate. The HU value was measured by placing the largest elliptical ROI at the vertebral body. The ROI was chosen to include as much trabecular bone as possible and to avoid cortical bone and heterogeneous areas, such as cortical bone margins, osteophytes, and osteosclerosis. The average of HU values calculated from the three ROIs was regarded as the HU for the individual vertebral body. (Fig. 1).

### Measuring TMC subsidence

**Fig. 2:**
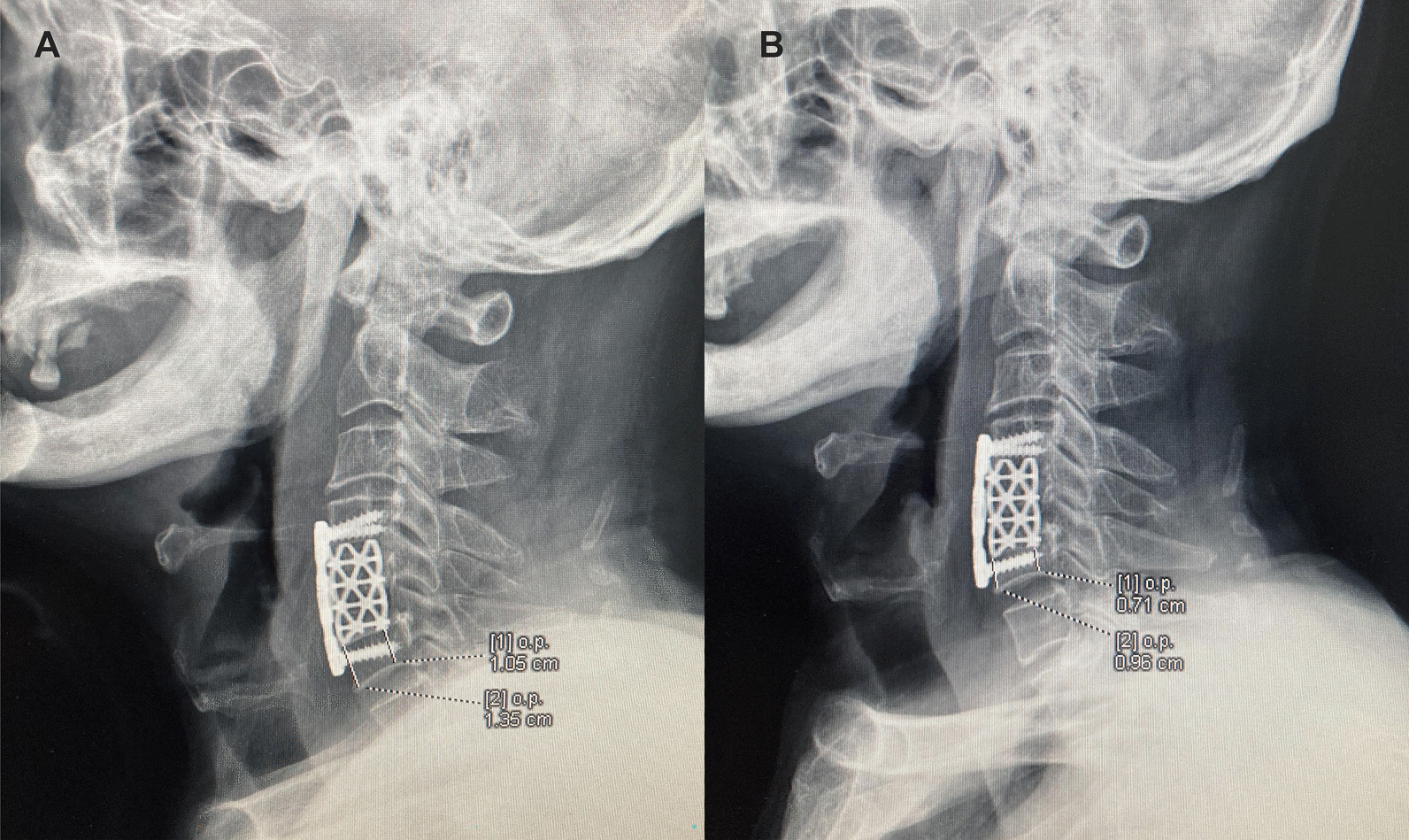
Compare immediately (**A**) and within 3 months (**B**) after surgery at lateral X-ray to calculate the TMC subsidence

Lateral radiographs taken with the patient in a standing position were obtained before the operation, immediately after operation and within 3 months after surgery. Lateral X-ray were used to calculate the TMC subsidence, and early TMC subsidence was diagnosed when the loss of any height of the anterior or posterior edges of the vertebral body in the fusion segment was greater than 3 mm within 3 months after surgery compared with that immediately after operation [21, 22]. The patients were divided into 2 groups according to the subsidence (Fig. 2).

### Clinical assessment

Clinical parameters included the pain visual analog scale (VAS), Japanese Orthopedic Association (JOA), and Neck Disability Index (NDI) scores. (1) VAS was an 11-point numeric rating scale ranging from zero (no pain) to ten (worst pain imaginable). (2) The JOA scale consisted of 3 categories: exercise, sensation, and bladder function. The total score was 17 points. (3) The NDI consisted of a 100-point scale. These are used both before surgery and after surgery. After discharge from the hospital, the patients had regular follow-ups conducted by the corresponding author.

### Radiological assessment

**Fig. 3:**
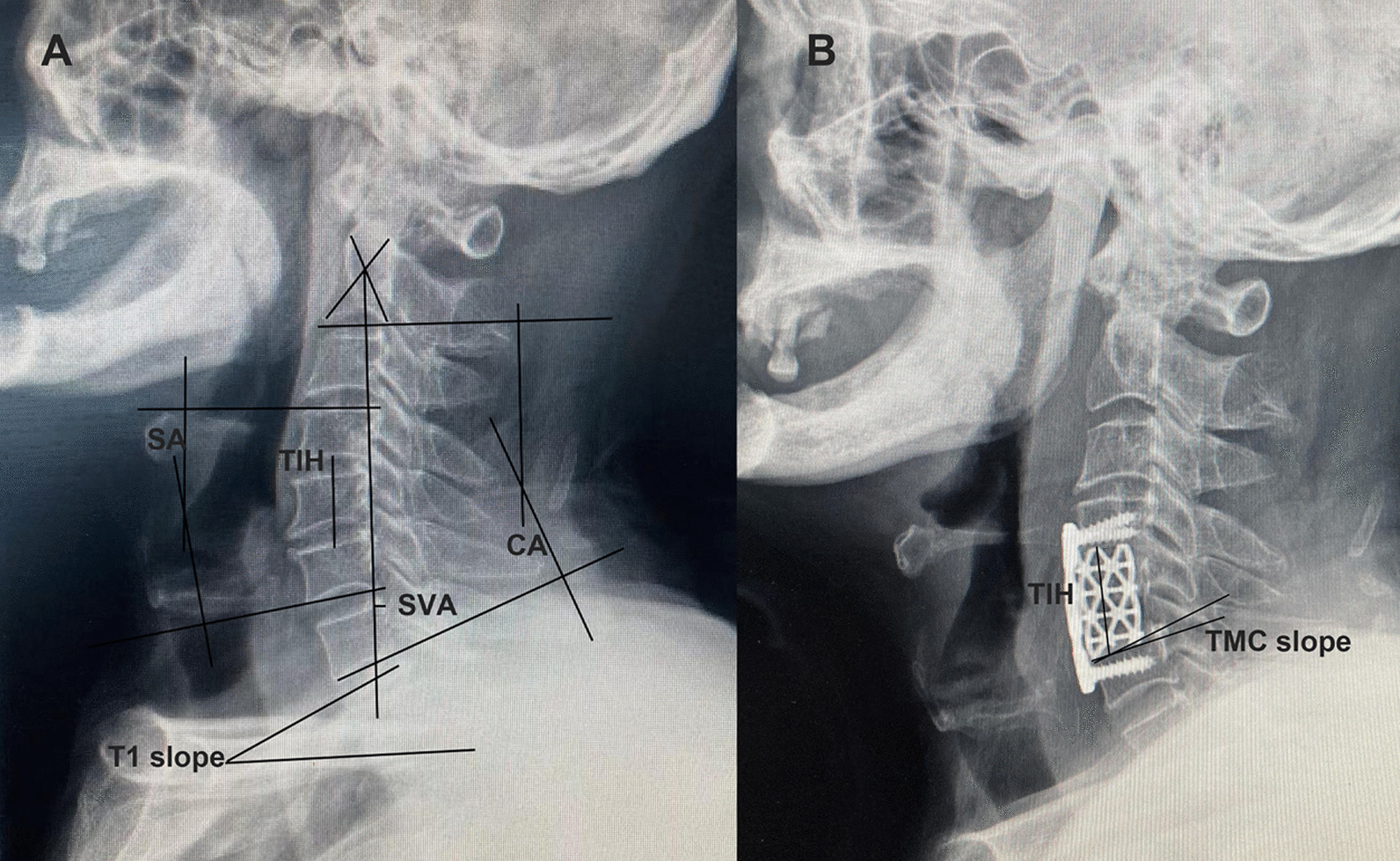
Measurement of the preoperative (**A**) and postoperative (**B**) radiologic parameters. Included the C2-7 Cobb angle (CA), segmental angle (SA), T1 slope, C2-7 sagittal vertical axis (SVA), total intervertebral height (TIH), and TMC slope

Radiologic parameters on plain radiographs included the C2-7 Cobb angle (CA), segmental angle (SA), T1 slope, C2-7 sagittal vertical axis (SVA), total intervertebral height (TIH), and TMC slope. The CA was defined by the Cobb angle formed between the lower endplate of C2 and C7. The SA was defined as the angle between the borders of endplates above and below the affected segment. The T1 slope was defined as the angle between the extension of the upper endplate of the T1 vertebral body and the horizontal line. The SVA was defined as the distance between a vertical line from a vertical line in the center of the C2 vertebral body to the posterior superior corner of the C7 vertebral body. TIH was measured as the distance from the midpoint of the superior endplate of the upper vertebral body to the midpoint of the inferior endplate of the lower vertebral body spanning the fusion. The TMC slope was defined as the angle between the lower end face of the TMC and the upper end plate of the lower vertebral body. Intervertebral distraction height was defined as postoperative TIH minus preoperative TIH. All measurements were performed by two independent investigators (Fig. 3).

### Statistical analysis

Statistical analysis was conducted using SPSS software (version 20, USA). After an agreement was reached between the observers, each parameter was independently measured twice by 2 orthopedic spine surgeons. Categorical variables are presented as absolute numbers and percentages, and continuous variables are presented as mean ± standard deviations. The Shapiro–Wilk test was used to verify the normal distribution of continuous variables. The independent sample t test was used for variables that followed a normal distribution. The Mann–Whitney U test was used for those not following normal distribution. Chi-squared test was used for categorical data (sex, Disease, surgical segment). Logistic regression analysis was used to identify the independent factors of TMC subsidence, and the results were presented as odds ratios (OR), with 95% confidence intervals. The receiver operating characteristics curve (ROC) was used to evaluate the value of predicting TMC subsidence, and the area under the curve (AUC) was calculated. The most appropriate threshold (cutoff value) of HU with higher sensitivity and specificity was also established using the ROC curve. *P* < 0.05 was considered to indicate a statistically significant difference.

## Results

### Demographic characteristics

**Fig. 4:**
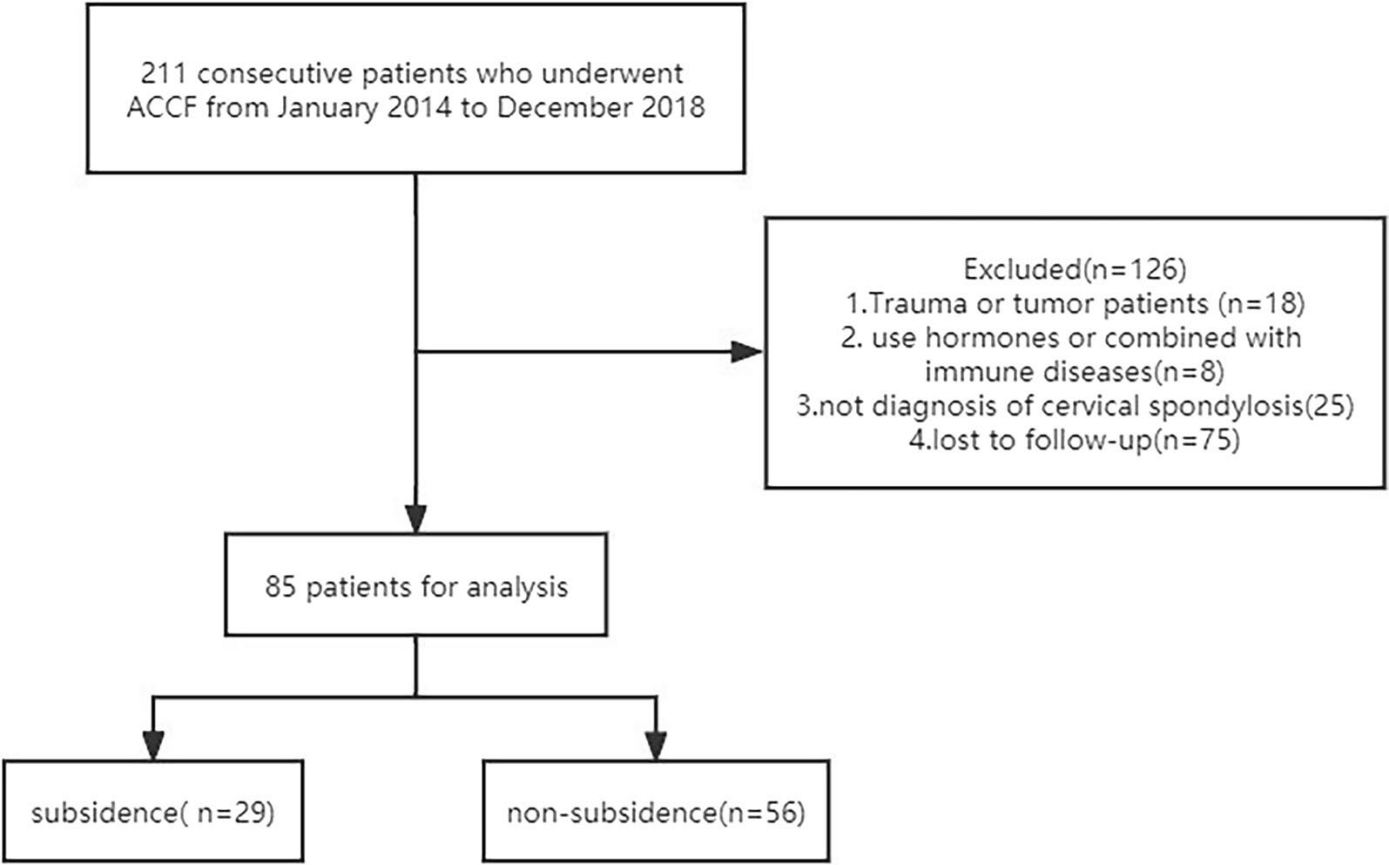
A flowchart of patients included in the study

We reviewed 211 patients who underwent ACCF with a TMC between January 2014 and December 2018, and a total of 85 patients met the inclusion criteria (Fig. 4). All the patients were diagnosed and divided into subsidence group (29 patients, 34.1%) and non-subsidence group (56 patients, 65.9%). In the subsidence group, the subsidence range was 3.0–6.4 mm (3.57 ± 1.01), the subsidence segment occurred in 5 cases in C5, 16 cases in C6, and 8 cases in C7. TMC subsidence occurred in 18 patients at 1-month postoperative, 4 patients at 2-month postoperative, and 7 patients at 3-month postoperative. Postoperative lateral cervical X-ray was taken at 1 day and 1.2.3 months and the final follow-up after surgery. The mean patient age was 59.01 ± 7.97 years old (range 45–82 years), and compared with the non-subsidence group (57.54 ± 9.84), the subsidence group age (63.09 ± 8.49) was significantly higher (*P* = 0.012). But there were no significant differences in sex, BMI, diabetes, hypertension, coronary heart disease, disease type, surgical segment, merge ACDF, hospital stay, surgery time, and blood loss between the two groups (Table 1).

**Table 1:** Demographic and surgery characteristics

Variable	Both cohorts	Subsidence	Non-subsidence	*p* value
No. of patients	85	29	56	
Age (yrs)	59.01 ± 7.97	63.09 ± 8.49	57.54 ± 9.84	0.012
Gender				0.996
male	41	14	27	
female	44	15	29	
BMI (kg/m^2^)	25.16 ± 3.22	25.35 ± 3.46	25.06 ± 3.11	0.701
Diabetes				0.240
yes	20	9	11	
no	65	20	45	
Hypertension				0.658
yes	21	8	13	
no	64	21	43	
Coronary heart disease				0.319
yes	8	4	4	
no	77	25	52	
Disease type				0.773
myelopathy	67	23	44	
radicular	7	3	4	
mixed	11	3	8	
Surgical segment				0.597
C4	12	5	7	
C5	43	16	27	
C6	30	8	22	
Merge ACDF				0.285
Yes	30	8	22	
No	55	21	34	
Hospital stays (Day)	11.11 ± 3.44	10.55 ± 2.31	11.39 ± 3.89	0.288
Surgery time (min)	105.45 ± 28.32	105.69 ± 33.16	105.32 ± 25.78	0.955
Blood loss (ml)	130.71 ± 134.98	124.48 ± 124.17	133.93 ± 141.24	0.762

### Clinical outcomes

The JOA, VAS, and NDI scores significantly improved in both groups immediately after the operation, 3 months after the operation and at the final follow-up compared with preoperative. However, there were no significant differences in the JOA, VAS, and NDI scores between the two groups in preoperative, 3 months after the operation, and at the final follow-up (Table 2).

**Table 2:** Clinical parameters

Variable		Total	Subsidence	Non-subsidence	*p* value
Preoperative	VAS	5.02 ± 1.20	5.07 ± 1.19	5.00 ± 1.22	0.804
	JOA	7.48 ± 1.05	7.38 ± 1.05	7.54 ± 1.06	0.520
	NDI	28.93 ± 5.54	28.48 ± 6.09	29.16 ± 5.28	0.596
Postoperative	VAS	2.22 ± 0.88*	2.17 ± 0.66	2.25 ± 0.98	0.666
	JOA	13.33 ± 1.37*	13.14 ± 1.16	13.43 ± 1.48	0.359
	NDI	15.96 ± 3.03*	15.93 ± 3.27	15.98 ± 2.92	0.942
3 months after the operation	VAS	2.19 ± 0.63*	2.27 ± 0.65	2.14 ± 0.62	0.357
	JOA	13.20 ± 1.02*	12.97 ± 0.94	13.32 ± 1.05	0.128
	NDI	15.26 ± 2.22*	15.48 ± 2.40	15.14 ± 2.13	0.506
The final follow-up	VAS	2.08 ± 0.64*	2.14 ± 0.58	2.05 ± 0.67	0.568
	JOA	13.16 ± 0.97*	12.90 ± 0.98	13.30 ± 0.95	0.067
	NDI	15.06 ± 1.84*	15.31 ± 1.87	14.93 ± 1.82	0.368

^*^P < 0.05 compared with preoperative

### Radiological outcomes

Between the subsidence and non-subsidence groups, there were significant differences in the intervertebral distraction height (*P* = 0.006). But there was no difference in preoperative, postoperative, and 3 months after operation in CA, SA, SVA, T1 slope, and TMC slope and their change between the two groups (Table 3).

**Table 3:** Radiographic parameters

	Variable	Subsidence	Non-subsidence	*p* value
	Intervertebral distraction height (cm)	0.49 ± 0.20	0.35 ± 0.21	0.006
Preoperative	CA (°)	14.14 ± 10.64	11.43 ± 9.46	0.234
	SA (°)	7.86 ± 5.20	7.20 ± 4.19	0.525
	SVA (cm)	2.15 ± 0.86	1.92 ± 0.82	0.236
	T1 slope (°)	24.90 ± 5.88	23.86 ± 6.89	0.491
Postoperative	CA (°)	16.52 ± 8.17	15.64 ± 8.76	0.657
	SA (°)	11.03 ± 6.15	10.14 ± 5.24	0.486
	SVA (cm)	2.22 ± 1.01	1.92 ± 0.91	0.177
	T1 slope (°)	25.90 ± 5.70	24.32 ± 6.73	0.202
	TMC slope(°)	10.62 ± 8.59	8.93 ± 6.38	0.307
3 months after the operation	CA (°)	15.03 ± 8.90	14.13 ± 8.63	0.650
	SA (°)	9.48 ± 5.38	9.05 ± 5.49	0.732
	SVA (cm)	2.18 ± 0.88	2.02 ± 0.83	0.428
	T1 slope (°)	25.24 ± 4.63	23.11 ± 5.10	0.063
	TMC slope (°)	11.83 ± 9.35	9.48 ± 6.43	0.177
Pre- and Postoperative	Change in CA	5.69 ± 3.43	7.46 ± 5.18	0.063
	Change in SA	3.52 ± 3.28	4.52 ± 3.24	0.183
	Change in SVA	0.63 ± 0.46	0.69 ± 0.51	0.624
	Change in T1 slope	2.93 ± 2.42	3.21 ± 2.65	0.632
Post- and 3 months after the operation	Change in CA	4.59 ± 3.98	4.88 ± 3.87	0.966
	Change in SA	3.76 ± 3.08	3.48 ± 2.12	0.747
	Change in SVA	0.55 ± 0.57	0.56 ± 0.45	0.628
	Change in T1 slope	3.69 ± 3.76	3.75 ± 3.11	0.937
	Change in TMC slope	4.03 ± 4.94	2.16 ± 1.96	0.058

### HU values of vertebral body and endplate

The HU values of both the upper vertebral body and endplate and the lower vertebral body and endplate in the subsidence group were lower than those in the non-subsidence group (lower and upper vertebral HU value: *P* < 0.0001; HU values of lower and upper end plate: *P* = 0.004) (Table 4).

**Table 4:** HU values of the vertebral body and endplate

Variable	Subsidence	Non-subsidence	*p* value
HU value of upper vertebral body	326.84 ± 64.76	423.56 ± 82.36	*P* < 0.0001
HU value of lower endplate of the upper vertebral body	492.93 ± 92.61	553.27 ± 85.93	0.004
HU value of lower vertebral body	251.44 ± 61.36	346.33 ± 71.01	*P* < 0.0001
HU value of upper endplate of the lower vertebral body	406.28 ± 119.92	475.70 ± 93.68	0.004

### Risk factors for subsidence

Univariate analysis showed that there were significant differences in age, intervertebral distraction height, HU value of upper and lower vertebral body and endplate between the subsidence and non-subsidence groups (*P* < 0.05). According to binary logistic regression analysis, independent risk factors were only HU value of the lower vertebral body (*P* = 0.008) (Table 5).

**Table 5:** Using binary logistic regression analysis to judge independent risk factors

Variable	B	*p* value	OR	95% CI
Age	0.007	0.835	1.007	0.942–1.077
Intervertebral distraction height	2.278	0.104	9.757	0.628–151.553
HU value of upper vertebral body	− 0.005	0.444	0.995	0.981–1.008
HU value of lower endplate of the upper vertebral body	− 0.003	0.609	0.997	0.988–1.007
HU value of lower vertebral body	− 0.026	0.008	0.975	0.956–0.993
HU value of upper endplate of the lower vertebral body	0.008	0.133	1.008	0.998–1.017

### ROC curve analysis

**Fig. 5:**
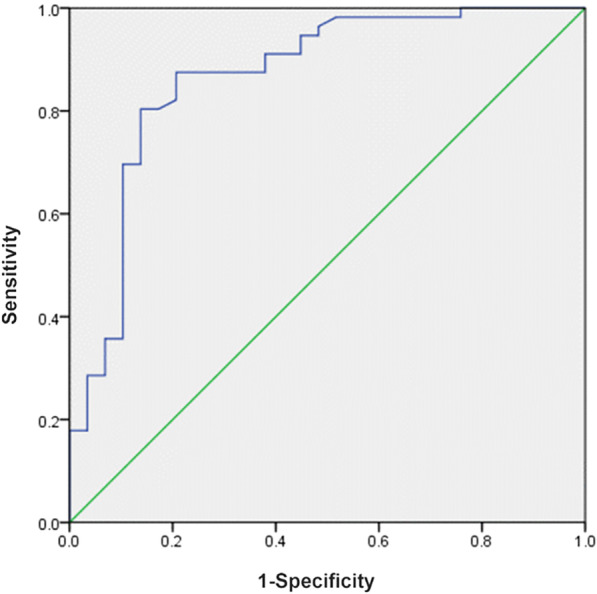
The ROC curves demonstrated that the areas under the curve (AUC) for HU value of lower vertebral body were 0.866 (*P* < 0.0001)

Using independent risk factors, we performed a ROC curve analysis. Sensitivity and specificity were calculated for the cutoff value (Table 6) and the area under the curve (AUC) (Fig. 5). The cutoff point was specified from the ROC curve using the optimal intersection of specificity and sensitivity. Based on the ROC curve, the cutoff point was 275 HU (sensitivity: 87.5%; specificity: 79.3%) at the lower vertebral body.

**Table 6:** Results of ROC analysis

Variable	AUC (95% CI)	*P* value	Cut off	Sensitivity	Specificity
HU value of lower vertebral body	0.866	< 0.0001	275.17	87.5%	79.3%

## Discussion

In this retrospective study, there were significant differences in age, Intervertebral distraction height, HU value of upper and lower vertebral body and endplate between the subsidence and non-subsidence groups. According to binary logistic regression analysis, the independent risk factor was HU value of the lower vertebral body, and the cutoff point was 275 HU (sensitivity: 87.5%; specificity: 79.3%). CT scans are routinely examined before ACCF, HU values may be used as a feasible predictor of the TMC subsidence after ACCF, which provides guidance for surgical planning.

Clinically, ACCF with TMC is widely used in the treatment of cervical degenerative disease, which has good clinical results. In our study, The JOA, VAS, and NDI scores significantly improved both in the subsidence and non-subsidence groups after operation. However, its shortcomings are becoming more and more obvious. TMC subsidence is the most important complication, such as broken screws and broken plates caused by the TMC subsidence. Therefore, it is particularly important to analyze the reasons for the TMC subsidence after surgery and how to take measures to prevent it. However, the measurement and standard of TMC subsidence have been controversial. Jon Bergen et al. [21] suggested that the postoperative intervertebral height decrease should be greater than 3 mm. Chen et al. [22] divided the subsidence of the titanium mesh into mild (1–3 mm) and severe (≥ 3 mm), and other studies have shown that about 80% of all patients with TMC subsidence occur within 3 months after surgery [23]. There are unavoidable errors in the process of imaging examination and measurement, and the error of setting the standard as 2 mm is relatively large. So in our study, we set the standard as the subsidence of the TMC measured by lateral cervical X-ray within 3 months was greater than 3 mm. A total of 29 occurred subsidence in our study, the subsidence rate was 34.1%, and the range was 3.0–6.4 mm.

TMC subsidence is presumed to be influenced by multiple factors. The potential risk factors consist of advanced age, osteoporosis, long segment, titanium mesh inclination angle, intervertebral distraction height, Sagittal imbalance, and so on [24, 25]. In our study, although there were significant differences in age between the two groups, patient age was not an independent risk factor. The reason may be that older patient always had lower BMD. Intervertebral distraction can restore the physiological curvature of the cervical spine and expand the area of the spinal canal. It is an important operation step in anterior cervical surgery. But many studies have reported excessive intervertebral distraction may cause the TMC to bear excessive intervertebral stress, leading to the occurrence of TMC subsidence [5]. Our study also suggested that there were significantly higher intervertebral distraction heights in subsidence groups. The old patients are often accompanied by osteoporosis, and among the risk factors for TMC subsidence, it is the most important. The loss of bone mass leads to a decreased bone density, destruction of trabecular bone structure, and a decreased in mechanical properties. Severe osteoporosis leads to stress fractures, screw loosening, and internal fixation failure after spinal surgery [26]. A biomechanical finite element analysis showed that with the increase in the degree of osteoporosis, the maximum stress on the upper and lower endplates of the fusion segment increased significantly, thus increasing the potential risk of implant subsidence [27]. In our study, the HU values of the upper and lower vertebral body and endplate in the subsidence group were significantly lower than those in the non-subsidence group, and those results indicate that BMD is a very important factor in TMC subsidence after ACCF.

The most commonly used parameter for the evaluation of BMD is DXA. DXA is an examination technique for two-dimensional measurement of BMD in areas such as the lumbar spine and hip joints, which cannot directly reflect the BMD of the cervical spine. However, when combined with degeneration, the bone density may increase due to abdominal aortic calcification, bone degeneration, fracture or osteophyte formation, etc., resulting in false negative results and missed diagnosis [13]. Moreover, DXA is not a routine inspection before cervical spine surgery. The BMD value of different vertebral bodies is significantly different. So DXA of the lumbar spine is not suitable for the cervical spine. Zou et al. [28] suggested that thresholds for osteoporosis based on HU values can be used as a complementary method to identify spinal osteoporosis in patients with lumbar degenerative diseases. At present, the use of conventional CT examination to measure vertebral HU value is gradually applied to the cervical spine, Lee et al. [19] found that the vertebral HU value can be a good alternative assessment to accurately reflect BMD in the cervical spine. Wang et al. [29] reported that lower preoperative HU values of the vertebral are associated with cage subsidence in single-level ACDF. Another study reported that lumbar BMD values were significantly correlated with cervical HU values; moreover, low HU values may lead to postoperative intervertebral height reduction [20].

There are many studies on the association between the low HU values and clinical effects in lumbar fusion surgery [17, 30–32]. However, clinical efforts to use HU values have been relatively limited in ACCF. In our study, the HU values of the upper and lower vertebral body and endplate in the subsidence group were significantly lower than those in the non-subsidence group, but only the HU value of the lower vertebral body was the independent risk factor for TMC subsidence. A biomechanical study shows that the compression resistance of the vertebrae is mainly borne by the cancellous bone. BMD of cancellous bone is more important for the assessment of bone quality [33]. The lower vertebral body bears more compressive stress, so the bone quality of the lower vertebral body has a greater impact on TMC subsidence. Cheng et al. [34] suggested that the extent of intervertebral space expansion, alignment of TMC, and poor BMD are the risk factors for subsidence. His results are similar to ours, but he measured the mean HU value of each vertebral body from C2 to C7. Since he did not take into account the effect of endplates, and ACCF surgery is often performed on the lower cervical spine, the HU value of the vertebral body adjacent to the surgical segment is more closely related to the subsidence of TMC. Moreover, a biomechanical study showed that overall mechanical strength and stiffness and HU in the superior endplate of the caudal vertebra were lower than those in the inferior endplate of the cranial in the same intervertebral disc. Because of the significant correlation between the cervical endplate HU and the mechanical properties of the endplate, a higher incidence of subsidence in the lower endplate was observed clinically [35]. We measured the HU value of the upper and lower vertebral body and endplate, included more BMD related factors, and concluded that the HU value of the lower vertebral body was an independent risk factor for TMC subsidence.

Some studies suggest that HU value measurement is a simple and rapid technique to assess bone quality. The range of HU values that are compatible with osteoporosis, and some studies have proposed different diagnostic thresholds values [36]. In our study, the HU value of the lower vertebral body based on the preoperative CT scan is a predictor factor of TMC subsidence after ACCF. According to the ROC curves, patients with a HU value < 275 at the lower vertebral of the surgical segment were likely to have TMC subsidence after ACCF. When performing ACCF, attention should be paid to the screening of risk factors for TMC subsidence in patients. Patients with severe osteoporosis and low bone density should choose this operation with caution. When surgical treatment is necessary, braces such as cervical collars should be worn for a long time after surgery, and it can limit the excessive activity of the cervical spine and decrease the occurrence of TMC subsidence.

This study has several limitations. First, this was a retrospective study, and the follow-up period was short. Second, this study included only 85 patients, limiting the ability of the multivariate analysis to identify statistically significant risk factors for subsidence. Third, while the present study identified the HUs of the lower vertebral body as a risk factor for subsidence, we did not conduct a DXA examination, which is no proven association between DXA and CT values in the present study. Finally, there may be errors in measuring TMC subsidence using lateral cervical spine radiography instead of CT, and we only study the early not the longtime relationship between TMC subsidence and function change in patients. Therefore, the results of this study should be interpreted with caution, and further research is required to confirm our findings.

## Conclusions

In summary, patients with lower vertebral body HU values are at a significantly higher risk of TMC subsidence in the early postoperative period after ACCF. Surgeons should choose the surgical approach carefully and inform patients about the risk of postoperative TMC subsidence, especially those lower vertebral body’s HU value of less than 275.

## Data Availability

All data generated or analyzed during this study are included in this published article.

## Abbreviations

HU: Hounsfield units
CT: Computed tomography
TMC: Titanium mesh cage
ACCF: Anterior cervical corpectomy and fusion
BMD: Bone mineral density
DXA: Dual-energy X-ray absorptiometry
ACDF: Anterior cervical discectomy and fusion
ROI: Region of interest
PACS: The picture archiving and communication system
VAS: Visual analog scale
JOA: Japanese Orthopedic Association
NDI: Neck Disability Index
CA: C2-7 Cobb angle
SA: Segmental angle
SVA: C2-7 sagittal vertical axis
TIH: Total intervertebral height
